# Supplementary material for: Sensory Appeal and Routines Beat Health Messages and Visibility Enhancements: Mixed-Methods Analysis of a Choice-Architecture Intervention in a Workplace Cafeteria
Source: Nutrients. 2022 Sep 10;14(18):3731. doi: 10.3390/nu14183731 (PMC9505513; doi:10.3390/nu14183731)
Supplement: Supplementary file 1 [file nutrients-14-03731-s001.zip › nutrients-1880256-supplementary.pdf]

Supplementary material

Heart-food = food items that met the product category-specific nutrition criteria of the Heart symbol.

Supplementary Table S1. Objects of interest on serving line stretches #1–2. During the intervention, heart-food items featured Heart symbols that were considered objects of interest per se.

Serving line	Weekday	Category	Object of interest	Heart-food
Stretch #1	all	Heart-symbol material [1]	”Follow the heart”-poster (A3), front of pillar	not applicable
	all	Heart-symbol material [1]	Heart label on a salad bar notice (A4), front of pillar	not applicable
Stretch #2	all	Heart-symbol material [1]	”A sign of good food”-poster (A3), back of pillar	not applicable
	all	Heart-symbol material [1]	Heart label on a salad bar notice (A4), back of pillar	not applicable
	all	Heart-symbol material [1]	Salad bar sign (A6) with Heart label, back of pillar	not applicable
	all	snack	Fruits	Yes
	all	snack	Blueberry quark	Yes
	all	snack	Sandwiches	No
	all	snack	Yoghurts	No
	all	snack	Muesli bars	No
	all	snack	Nut and dried fruit mix	No
	all	salad bar	Salad components as a whole (n = 18–19 per day)	Yes [2]
	all	salad bar	Salad dressings	Yes and no
	Monday	warm course	Steamed vegetables	Yes
	Monday	warm course	Boiled potatoes	No [3]
	Monday	warm course	Wholegrain rice	No [3]
	Monday	warm course	Chicken rissoles	Yes
	Monday	warm course	Curry sauce for chicken rissoles	Yes
	Monday	warm course	Bean and vegetable sauce	Yes
	Monday	warm course	Pureed vegetable soup with cheese	Yes
	Monday	warm course	Beef wok	No
	Tuesday	warm course	Steamed vegetables	Yes
	Tuesday	warm course	Boiled potatoes	Yes
	Tuesday	warm course	Beetroot croquettes	Yes
	Tuesday	warm course	Potato wedges	No
	Tuesday	warm course	Hamburgers	No
	Tuesday	condiment	Chilli-mayonnaise	No
	Tuesday	warm course	Salmon soup	No [4]
	Tuesday	dessert	Lingonberry quark	No
	Wednesday	warm course	Steamed vegetables	Yes
	Wednesday	warm course	Boiled potatoes	Yes
	Wednesday	warm course	Wholegrain rice	Yes
	Wednesday	warm course	Fish cutlets with Cheddar cheese	Yes
	Wednesday	condiment	Yoghurt dressing and lemon for fish	No
	Wednesday	warm course	Vegetarian curry	Yes
	Wednesday	warm course	Chorizo casserole	No
	Wednesday	warm course	Spinach soup and boiled eggs	No
	Thursday	warm course	Steamed vegetables	Yes
	Thursday	warm course	Broad bean and pasta casserole	Yes
	Thursday	warm course	Pureed sweet potato soup with chilli	Yes
	Thursday	warm course	Mashed potatoes	No
	Thursday	warm course	Oven-baked sausages with grated cheese	No
	Thursday	warm course	Chicken tortillas	No
	Thursday	condiment	Taco sauce for tortillas	No
	Thursday	condiment	Salsa for tortillas	No
	Thursday	condiment	Sour cream for tortillas	No
	Thursday	dessert	White chocolate mousse	No
	Friday	warm course	Steamed vegetables	Yes
	Friday	warm course	Boiled potatoes	Yes
	Friday	warm course	Fish sauce with lemon	Yes
	Friday	warm course	Vegetarian moussaka	Yes
	Friday	warm course	Beef burgers filled with pepper sauce	No
	Friday	warm course/condiment	Gravy/sour cream for beef burgers	No
	Friday	warm course	Blue cheese soup	No
	Friday	dessert	Fruit salad and vanilla sauce	No

[1] In place only during intervention. [2] All salad components were considered heart-food items because the implementation of Heart labels and the analysis of eye-tracking data were not feasible at the level of individual salad components. [3] Boiled potatoes and wholegrain rice met the nutritional criteria of the Heart symbol but received no symbols on intervention week's Monday. Hence, on Mondays these foods were categorised as non-heart-foods and on other days as heart-foods. [4] Salmon soup met the criteria of the Heart symbol only during the control condition. Hence, the soup received no Heart symbol during intervention and was categorised as non-heart-food.

Supplementary Table S2. Definitions for the areas of interest (AoI) of the objects of interest on serving line stretches #1–2.

Category	Object of interest	Area of interest
Heart-symbol material [1]	Posters, notices, stickers, and signs	Outlines of Heart-symbol posters, notices, stickers, or signs added on the serving line
Snack	Fruit	Outlines of fruit and their serving platter
Snack	Sandwiches, blueberry quark, yoghurts, muesli bars, nut and dried fruit mix	Outlines of food packages, including possible serving platters
Salad bar	Salad components	Combined outlines of the serving dishes of available salad components, including spaces between adjacent serving dishes. These outlines defined the AoI also when the serving dishes had only little food left and the bottoms and/or insides of the dishes were exposed. If food items rose above the tops of the serving dishes, the foods themselves defined the top of the AoI.
Salad bar	Salad dressings	Outlines of salad dressing bottle.
Warm course	Warm courses	Outlines of the serving dishes of individual warm course items, excluding lids and hoods and spaces between adjacent serving dishes. These outlines defined the AoIs also when the serving dishes had only little food left and the bottoms and/or insides of the dishes were exposed. If food items rose above the tops of the serving dishes, the foods themselves defined the tops of the AoIs.
Condiment	Sauces for warm courses	Outlines of serving dishes
Dessert	Dessert items	Outlines of serving dishes

[1] In place only during intervention.

Supplementary Table S3. Perceived influences on food choices with example quotes from interview data. The influences are presented in a descending order according to the total number of individuals that mentioned each influence.

Influence	Description	Example quotes
Sensory appeal	The look, taste, or texture of food; less-specified preferences	The appearance of food; attractiveness matters.<br>Mental image of flavour. If there is a choice after that, I choose the healthier option. Flavour is nevertheless the most important thing.<br>Rye bread is important, but if it is too hard, I don't take it.<br>Other options were not pleasant.
Healthiness	Healthiness in general, meal composition, nutritional content, specific dietary guidelines, the Heart symbol	Healthiness.<br>That there are vegetable, protein, and carbohydrate sources to form a healthy plate model.<br>Rich in protein.<br>I aim to eat fish when it is available, due to guidelines.<br>Sometimes I use the Heart symbol to support my choices. For example, now I took milk instead of juice due to the Heart symbol.
Familiarity	Habitual, familiar, or traditional choices	What I chose is basic food I usually eat.<br>Tortillas have not always been very good. I considered this a safer choice.<br>I prefer rather traditional foods.
Particular foods	Particular food, food group, or meal component	Salad table, fresh produce.<br>I favour fish when it's available.
Variation	The importance of having a wide variety of options to choose from, desire to choose diverse foods or foods different to those eaten elsewhere, curiosity to try new foods, motivation towards specialties rarely served	Variety; that there are diverse options.<br>A balanced meal.<br>No red meat, I eat it otherwise so much that I try to avoid it.<br>Seemed interesting. What is mifu *(a dairy-based meat alternative)* like?<br>Rarely served. I planned to have salad but noticed the hamburgers and changed my mind in the nick of time.
Weight control	Weight management, lightness, conscious regulation of portion sizes	I look at what I eat in relation to *(energy)* consumption. With heavy workout, it's ok to eat more heavily.<br>That the food is not very fatty.<br>I may have taken a bit too much of the carbohydrate accompaniment, I paid attention to that after portioning. I considered whether to take two fish cutlets. I took one.
Menu	The menu online or at the restaurant.	I checked the menu at the entrance and practically made my decision there. I still compared the main courses at the serving line, yet kept my original choice.

Influence	Description	Example quotes
Satiety	Foods that fill the stomach and take away the hunger	That the food is filling.<br>I often take the Heart label option but chose sausage today because I'm very hungry.
Mood	Foods that help to cope with work/personal life commitments, choices made based on a feeling.	I try to choose a fairly light lunch that doesn't make me tired in the afternoon.<br>Soup would have been enough, but I have a long and physically active day ahead.<br>*(I made my choice)* based on vibes, what I fancied today.
Special diet	Special dietary requirements	I need gluten free food.<br>I'm a vegetarian.
Food quality	The type of food or ingredients	The quality of food.<br>On the bread table, *(I checked)* whether there was fresh bread.
Convenience	Focus on time needed to acquire or eat food.	*(It is important)* to get the food rather quickly.<br>*(I made my choice)* based on which food is quick to eat.
Price	Affordable prices, price-quality ratio	Affordable price is important as well as getting healthy food at a reasonable price.<br>Price-quality ratio.
Season	New Years' resolutions, weather outside	The choice was based on a New Year's lifestyle change; the proportion of salad is greater than before.<br>The soup was tempting due to the cold weather.
Social influence	Recommendations of the cafeteria staff, experiences of other customers	Sometimes I ask recommendations from the restaurant staff.<br>I happened to hear that the steaks are very peppery. That's why I chose the fish.
Natural content	Processing	In principle, I do not eat highly processed food.
Ethical concern	Food origin	With the side dish, I pay attention to whether it is local.
